# Well-Being of Canadian Adolescents Who are Blind or Have Low Vision: A Cross-Sectional Study

**DOI:** 10.1177/0145482X261441583

**Published:** 2026-06-17

**Authors:** Aadesh Nunkoo, Christopher Power, Michael McIsaac

**Affiliations:** 1 School of Mathematical and Computational Sciences, 2359 University of Prince Edward Island, Charlottetown, Canada

**Keywords:** adolescents, blind or low vision, multiple disabilities, well-being, mental health

## Abstract

**Introduction:** This study investigates the well-being of Canadian adolescents who are blind or have low vision compared to their peers without visual disabilities. **Methods:** We analyzed cross-sectional data from the 2017–2018 Canadian Health Behaviour in School-aged Children survey. Our sample consisted of 19,702 Canadian adolescents aged 11 to 15 years, including 401 who self-reported a diagnosis of blindness or low vision, while the remaining 19,301 reported no visual disabilities. Chi-square tests assessing differences in proportions pertaining to subjective and psychological well-being, self-rated health, life satisfaction, and loneliness between these two groups were conducted. Binary and ordinal logistic regression models were used for multivariate analysis. **Results:** Canadian adolescents with blindness or low vision reported significantly diminished life satisfaction and a significantly higher prevalence of loneliness. They experienced mental illness and depressive symptoms at a significantly greater rate than their peers without visual disabilities. Well-being measures for adolescents who are blind or have low vision and *multiple disabilities,* which we defined as a participant who reported at least one additional disability other than blindness or low vision, were significantly more compromised. **Discussion:** Blindness or low vision was associated with lower well-being outcomes, including life satisfaction and loneliness, as well as poorer mental health among Canadian adolescents. The presence of multiple disabilities exacerbated the already lower levels of well-being outcomes experienced by adolescents with visual disabilities. **Implications for Practitioners:** Practitioners should recognize the well-being disparities experienced by Canadian adolescents who identify as being blind or having low vision, particularly those with multiple disabilities, and develop targeted interventions to address these challenges. Increasing access to specialized mental health support and promoting inclusive school environments are essential for improving the well-being of adolescents with visual disabilities.

Well-being, in general, is lower for disabled people (Bellia, 2021; Emerson et al., 2009; Emerson & Llewellyn, 2023; Savage et al., 2014). Although the reasons for this lower well-being are varied, issues such as loneliness and social isolation are also increased in communities of disabled people (Emerson et al., 2021; Gilmore & Cuskelly, 2014; Macdonald et al., 2018), as is the prevalence of depression and anxiety (Bhattacharjee & Chhetri, 2014; Honey et al., 2011; Lindén-Boström & Persson, 2015).

Adolescents who are blind or have low vision experience lower well-being, including poorer mental health outcomes and lower life satisfaction (Augestad, 2017; Boadi-Kusi et al., 2023; Choi et al., 2018; Habib & Irshad, 2018; Li et al., 2022; Oliveira et al., 2018; Pinquart & Pfeiffer, 2011). Additionally, adolescents with visual impairments are at an increased risk of social isolation and tend to experience higher levels of loneliness (Beteinaki, 2019; Chiracu & Buică-Belciu, 2023; Stanimirović et al., 2017).

However, research is often grounded in small numbers of adolescents with visual disabilities. For example, in the 17 studies reviewed by Augestad (2017), each had a relatively small number of participants with visual impairments, with the maximum being 182 (Pinquart & Pfeiffer, 2013); the study by Habib and Irshad (2018) used only 40 participants; and Oliveira et al. (2018) used only 18 individuals who are blind or have low vision. Additionally, some data are repurposed for analyses; for example, in the comprehensive review conducted by Li et al. (2022), the majority of articles that had a large number of participants with visual impairments were related to prescreening for military service.

Most studies with large numbers of participants who are blind or have low vision involve adult populations and primarily focus on a single disability (Brunes et al., 2019; Brunes & Heir, 2020; Schuster et al., 2018; Zhang et al., 2013).

**☑ Earn CEs Online**

by answering questions on this article.

For more information, visit:

https://www.aerelearning.org/

There is little research that looks at how the presence of other disabilities that co-occur with visual disabilities influences outcomes in adolescents.

In this article, we answer the following two questions for the Canadian context:

- What is the well-being of adolescents who are blind or have low vision in comparison to their peers without visual disabilities?
- What is the effect of multiple disabilities on the well-being of adolescents with visual disabilities?

## Materials and Methods

### Data Source

Data for this study comes from the 2017–2018 Canadian Health Behaviour in School-aged Children (HBSC) survey, a cross-national study conducted in collaboration with the World Health Organization (WHO) that examines the well-being and health behaviors of adolescents aged 11 to 15 years (Inchley et al., 2020). Self-report questionnaires assessing mental health, health behaviors, and sociodemographic characteristics are administered in school classrooms and completed individually by students, either on paper or electronically (Craig, 2020).

The responsibility for making the survey available in an accessible form to accommodate participants with disabilities remains the responsibility of the participating schools. Previous studies related to HBSC focusing on disability have included analyses of the health outcomes of disabled adolescents in general and the relationship of those health outcomes to life satisfaction (Canha et al., 2016; Fridh et al., 2018).

The protocol and survey questions asked of Canadian students are developed through a broad-based consultation model alongside the Public Health Agency of Canada (PHAC), provincial and territorial Ministries of Health and Education through the Pan-Canadian Joint Consortium for School Health and the Canadian HBSC team (Craig, 2020). The General Research Ethics Board at Queen's University, and the Health Canada/PHAC granted ethics approval for the HBSC survey. After obtaining permission from all 10 provinces and three territories in Canada, active consent was obtained from school boards and individual schools. Passive consent was given by the participants and their parents or guardians (McIsaac et al., 2021).

The HBSC continuously develops and validates its research instruments as part of its quality assurance process, ensuring robust research conclusions. The study has demonstrated good reliability, and validation efforts are ongoing across member countries, with new instruments and items being developed for each survey cycle. Over the years, HBSC members have conducted and published validation studies on a wide range of topics (Inchley et al., 2018).

Our dataset included adolescents from 224 Canadian schools who participated in the study. The sample was designed to be nationally representative within Canada as a whole (McIsaac et al., 2021). Our study sample consisted of 19,702 participants aged 11 to 15 years, divided into two groups: 19,301 adolescents without visual disabilities and 401 participants who self-reported that they had been diagnosed with blindness or low vision. The blind or low vision group was further divided into two subgroups: 292 participants who reported only blindness or low vision, and 109 participants who reported at least one additional disability.

### Study Variables and Measures

#### Disability

Participants were asked to report diagnosed learning exceptionalities or special education needs through the following question: “If you have been diagnosed with a learning exceptionality or special education need, please indicate which one. (You may choose one answer, or more than one).” We considered the following response categories:

- I have not been diagnosed with a learning exceptionality or special education need (*n* = 11,192),
- autism or Asperger's syndrome (*n* = 197),
- behavior (*n* = 239),
- blind or low vision (*n* = 401),
- deaf or hard-of-hearing (*n* = 133),
- attention-deficit hyperactivity disorder (ADHD) or attention-deficit disorder (ADD) (*n* = 1026),
- intellectual disability (*n* = 26),
- language or speech impairment (*n* = 231),
- learning disability (*n* = 530),
- physical disability (*n* = 82), and
- other (*n* = 321).

#### Outcome Variables

Several key variables were used as well-being indicators in this study.

##### Subjective Well-Being

The WHO-5 well-being index was used to assess the general, subjective, and psychological well-being over the past two weeks. It includes the following five items: *I have felt cheerful and in good spirits*, *I have felt calm and relaxed*, *I have felt active and energetic*, *I have woken up feeling fresh and rested*, and *my daily life has been filled with things that interest me.* Participants are asked to rate each statement according to the following 6-point Likert scale: 1 = *all of the time*, 2 = *most of the time*, 3 = *more than half of the time*, 4 = *less than half of the time*, 5 = *some of the time*, 6 = *at no time*. The WHO-5 well-being index raw score ranges from 5 to 30. Studies on the WHO-5 well-being index's validity have shown strong clinometric, internal, and external validity due to its high sensitivity and specificity (Lukaschek et al., 2017; Topp et al., 2015). The responses were categorized into poor (5–10), fair (11–17), good (18–24), and very good (25–30) (Lukaschek et al., 2017).

##### Life Satisfaction

The HBSC survey uses Cantril's ladder, an efficient measure with high construct validity, to evaluate life satisfaction (Levin & Currie, 2014). The participants were shown a picture of a ladder with steps ranging from 0 (*worst possible life*) to 10 (*best possible life*) and were asked to indicate where they would place their lives at present. The responses were categorized into low life satisfaction (0–5), normal life satisfaction (6–7), and high life satisfaction (8–10) (Gazendam et al., 2020).

##### Self-Rated Health

Self-rated health of the participants was measured through the following question: “Would you say your health is …?” A four-point response was used with these categories: 1 = *excellent*, 2 = *good*, 3 = *fair*, and 4 = *poor*. The categories were dichotomized into Excellent or Good versus Fair or Poor.

##### Diagnosed Mental Illness and Depression

For diagnosed mental illness, participants were asked if they “… have been diagnosed with Mental Illness (e.g., depression, anxiety, bipolar disorder).” To assess depressive symptoms, participants were asked whether they “… ever felt so sad or hopeless almost every day for two weeks or more in a row that they stopped doing some usual activities (yes or no)” (McIsaac et al., 2023).

##### Loneliness

Students were asked to rate their agreement or disagreement with the statement, “I often feel lonely,” which was used to measure loneliness. The response categories were: 1 = *strongly agree*, 2 = *agree*, 3 = *neither agree nor disagree*, 4 = *disagree*, 5 = *strongly disagree*. The responses were dichotomized, and loneliness was defined as those reporting strongly agree or agree to the above statement (Brunes et al., 2019; Emerson et al., 2021; Favotto et al., 2019).

#### Demographics

Age was measured in years. Gender identity was recorded by asking the student which of these terms describes them: *male*, *female*, or *neither term describes me*. Racial or cultural background was obtained by asking the following question: “People living in Canada come from many different cultural and racial backgrounds. How do you describe yourself?” Participants described their family structure by indicating which adults they live with “… for the home where they live all or most of the time.” Subjective socioeconomic status was evaluated based on perceived family wealth (World Health Organization, 2016), which was assessed by asking participants to rate the following item: “How well off do you think your family is?” The scale rating consisted of five categories that ranged from *very well off* to *not at all well off* (Currie et al., 2008). This item has demonstrated strong test–retest reliability (Favotto et al., 2019) and has been used as an indicator of socioeconomic status in previous HBSC studies (Brook et al., 2024; Iversen & Holsen, 2008).

### Data Analysis

The study sample was described based on key sociodemographic variables. Differences in proportions of subjective and psychological well-being, self-rated health, life satisfaction, and loneliness among the group without visual disabilities and the blind or low vision group were evaluated using chi-square tests. Proportions were also compared between visually impaired adolescents without any additional disabilities and those with other disabilities, and 95% confidence intervals (CIs) for the differences in proportions were calculated. Adjusted *p* values were computed from binary logistic models for the outcomes loneliness, diagnosed mental illness, and depression. Ordinal logistic regressions were used to model subjective well-being, life satisfaction, and self-rated health. Statistical software R version 4.2.2 (R Core Team, 2022) was used for the analysis.

## Results

Our analysis included 19,702 participants, of whom 19,301 participants (98.0%) did not have visual disabilities, while 401 (2.0%) self-reported as having been diagnosed with blindness or low vision. Among the 401 adolescents who are blind or have low vision, 109 self-reported at least one additional disability: 9 (8.3%) had autism or Asperger's syndrome, 22 (20.2%) had behavioral difficulties, 19 (17.4%) were deaf or hard-of-hearing, 39 (35.8%) had ADHD or ADD, 1 (0.9%) had an intellectual disability, 24 (22.0%) had a language or speech impairment, 37 (33.9%) had a learning disability, 7 (6.4%) had a physical disability, and 11 (10.1%) selected other.

Table 1 presents the characteristics of the sample. Table 2 details the differences in well-being measures between adolescents without visual disabilities and those who identify as blind or as having low vision. Table 3 investigates the impact of multiple disabilities alongside blindness or low vision on the well-being of young people. In addition, the breakdowns of the survey results for the different well-being measures are summarized in terms of proportions in subsequent figures. The outcomes were categorized as presented in Tables 2 and 3 to maintain consistency with precedent (e.g., Emerson et al., 2021; McIsaac et al., 2023; Pierannunzio et al., 2022).

**Table 1:** Description of the 2017–2018 Canadian HBSC Sample of School-Aged Children With Blindness or Low Vision and Those Without

Variable	Group Without VisualDisabilities (*N* = 19,301)	Blind or Low VisionGroup (*N* = 401)
Grade		
5 and 6	3,886 (20.1%)	74 (18.5%)
7	4,417 (22.9%)	76 (19.0%)
8	4,346 (22.5%)	95 (23.7%)
9	4,585 (23.8%)	111 (27.7%)
10 and 11	2,066 (10.7%)	45 (11.2%)
Missing	1	0
Age in years, mean (*SD*)	13.7 (1.3)	13.8 (1.3)
Gender identity		
Male	9,223 (48.0%)	153 (38.3%)
Female	9,700 (50.5%)	233 (58.4%)
Neither term describes me	274 (1.4%)	13 (3.3%)
Missing	104	2
Racial or cultural background		
White	12,662 (66.7%)	213 (54.3%)
Black	665 (3.5%)	16 (4.1%)
Latin American	178 (0.9%)	5 (1.3%)
Indigenous (First Nations, Metis, or Inuit)	1,453 (7.7%)	23 (5.9%)
East and Southeast Asian	610 (3.2%)	10 (2.6%)
East Indian and South Asian	478 (2.5%)	21 (5.4%)
Arab and West Asian	262 (1.4%)	7 (1.8%)
Other (including mixed)	2,669 (14.1%)	97 (24.7%)
Missing	324	9
Subjective socioeconomic status		
Very well-off	4,196 (25.0%)	70 (20.6%)
Quite well-off	4,767 (28.4%)	99 (29.2%)
Average	6,285 (37.5%)	134 (39.5%)
Not very well-off	1,010 (6.0%)	27 (8.0%)
Not at all well-off	500 (3.0%)	9 (2.7%)
Missing	2,543	62
Family structure		
Mother and father	13,532 (71.8%)	264 (67.2%)
Mother and partner	1,012 (5.4%)	30 (7.6%)
Mother only	2,935 (15.6%)	66 (16.8%)
Father and partner	222 (1.2%)	8 (2.0%)
Father only	565 (3.0%)	10 (2.5%)
Other	592 (3.1%)	15 (3.8%)
Missing	443	8

*Note*. HBSC = Health Behaviour in School-aged Children.

**Table 2:** Well-Being Outcomes of the Group Without Visual Disability and the Blind or Low Vision Group

Outcome	Group Without VisualDisabilities (*N* = 19,301) (%)	Blind or Low VisionGroup (*N* = 401) (%)	Differencein Percentage [95% CI]	Chi-SquareTest Statistic	Bivariate*p* Value	Adjusted*p* Value^a^
Subjective well-being: good/very good	13,604 (75.4%)	234 (64.3%)	−11.1 [−16.1, –6.2]	23.77	<.0001	.0005
Life satisfaction: high	9,574 (51.9%)	138 (37.0%)	–14.9 [−19.9, –10.0]	32.60	<.0001	.0003
Self-rated health: good/excellent	15,347 (82.2%)	269 (70.1%)	–12.1 [–16.8, –7.5]	37.56	<.0001	<.0001
Diagnosed mental illness: yes	1,048 (5.4%)	66 (16.5%)	11.1 [7.4, 14.7]	89.58	<.0001	<.0001
Feeling sad or hopeless: yes	5,740 (31.6%)	197 (52.7%)	21.1 [16.0, 26.2]	75.10	<.0001	<.0001
Feeling lonely: yes	4,870 (26.5%)	171 (45.7%)	19.2 [14.1, 24.3]	69.10	<.0001	<.0001

a Binary and ordinal logistic regression models were fitted, and the corresponding *p* values adjusting for age, gender identity, racial or cultural background, socioeconomic status, and family structure were recorded. CI = confidence interval.

**Table 3:** Well-Being Outcomes of Adolescents Reporting Only Blindness or Low Vision and With Other Disabilities

Outcome	Blind Or LowVision Only (*N* = 292) (%)	Blind or Low Vision andOther Disabilities (*N* = 109) (%)	Difference inPercentage [95% CI]	Chi-SquareTest Statistic	Bivariate*p* Value	Adjusted*p* Value^a^
Subjective well-being: good/very good	182 (67.2%)	52 (55.9%)	–11.3 [–22.8, 0.3]	3.81	.0509	.0998
Life satisfaction: high	117 (41.9%)	21 (22.3%)	–19.6 [–29.8, –9.4]	11.58	.0007	.0011
Self-rated health: good/excellent	209 (73.9%)	60 (59.4%)	–14.5 [–25.3, –3.6]	7.40	.0065	.0085
Diagnosed mental illness: yes	31 (10.6%)	35 (32.1%)	21.5 [12.0, 30.9]	26.67	<.0001	<.0001
Feeling sad or hopeless: yes	131 (47.5%)	66 (67.3%)	19.9 [8.9, 30.9]	11.47	.0007	.0211
Feeling lonely: yes	119 (43.1%)	52 (53.1%)	10.0 [–1.5, 21.4]	2.88	.0896	.4957

a Binary and ordinal logistic regression models were fitted, and the corresponding *p* values adjusting for age, gender identity, racial or cultural background, socioeconomic status, and family structure were recorded. CI = confidence interval.

The age distribution was similar for both groups, with a mean age of 13.7 years (*SD* = 1.3) for participants without visual disabilities and 13.8 years (*SD* = 1.3) for those with blindness or low vision (see Table 1). The majority identified as female (50.5% of participants without visual disabilities, 58.4% of participants with visual disabilities), and reported White as a racial or cultural background (66.7% of participants without visual disabilities, 54.3% of participants with visual disabilities). A large proportion of participants reported their family wealth as “average” or “above average” (90.9% of participants without visual disabilities, 89.3% of blind and low vision participants). The majority lived with both a mother and father all or most of the time (71.8% of participants without visual disabilities, 67.2% of participants with visual disabilities).

**Figure 1:**
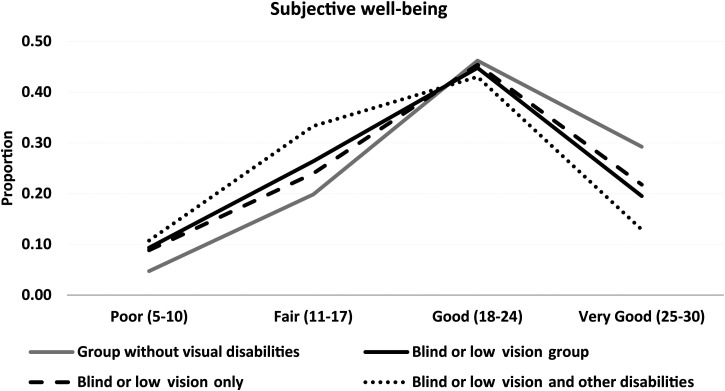
Proportion Breakdown of Categorized Well-Being WHO-5 Scores. WHO** **=** **World Health Organization

**Figure 2:**
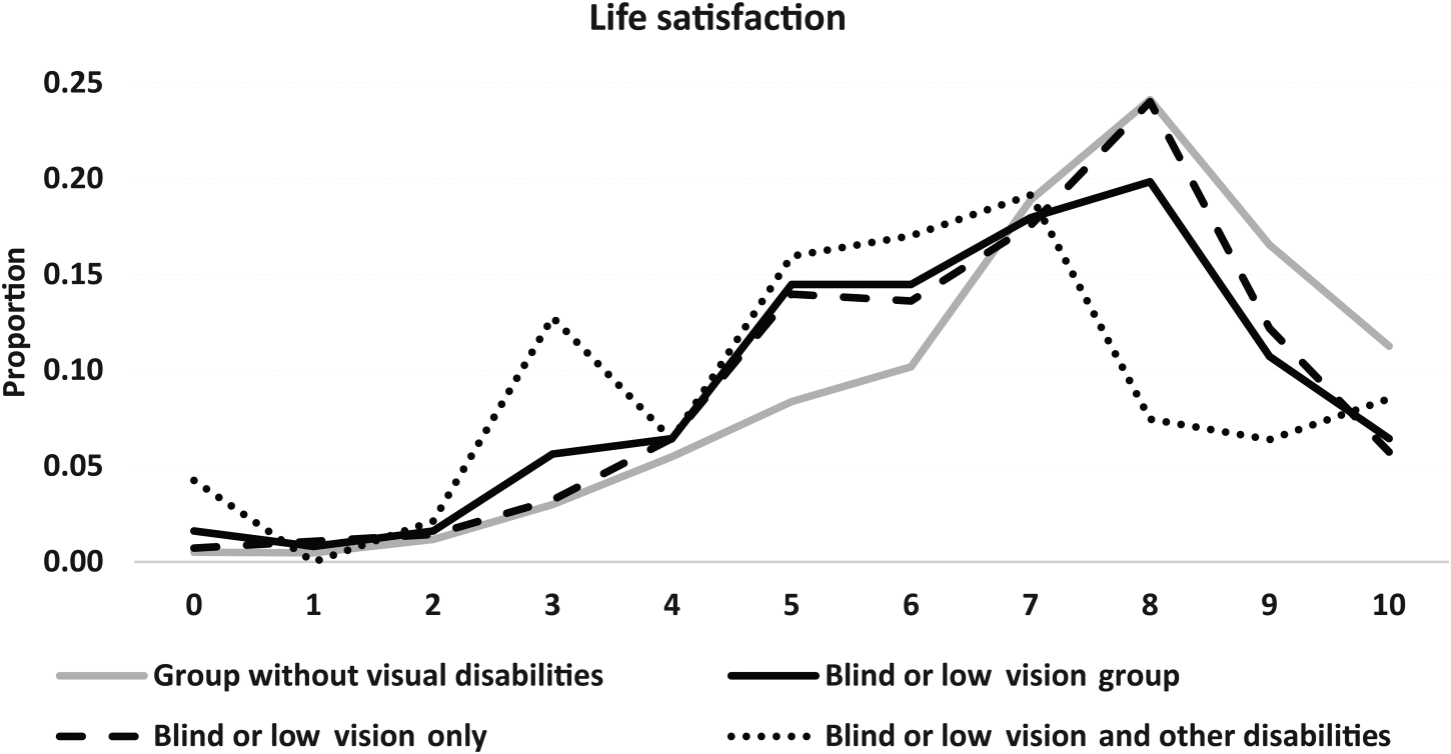
Proportion Breakdown of Life Satisfaction Responses

**Figure 3:**
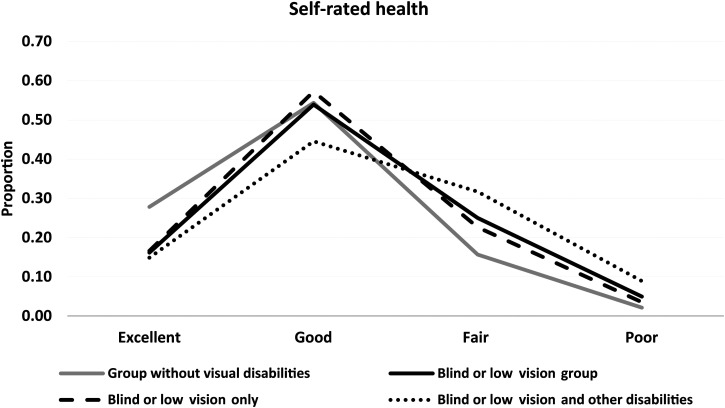
Proportion Breakdown of Self-rated Health Responses

**Figure 4:**
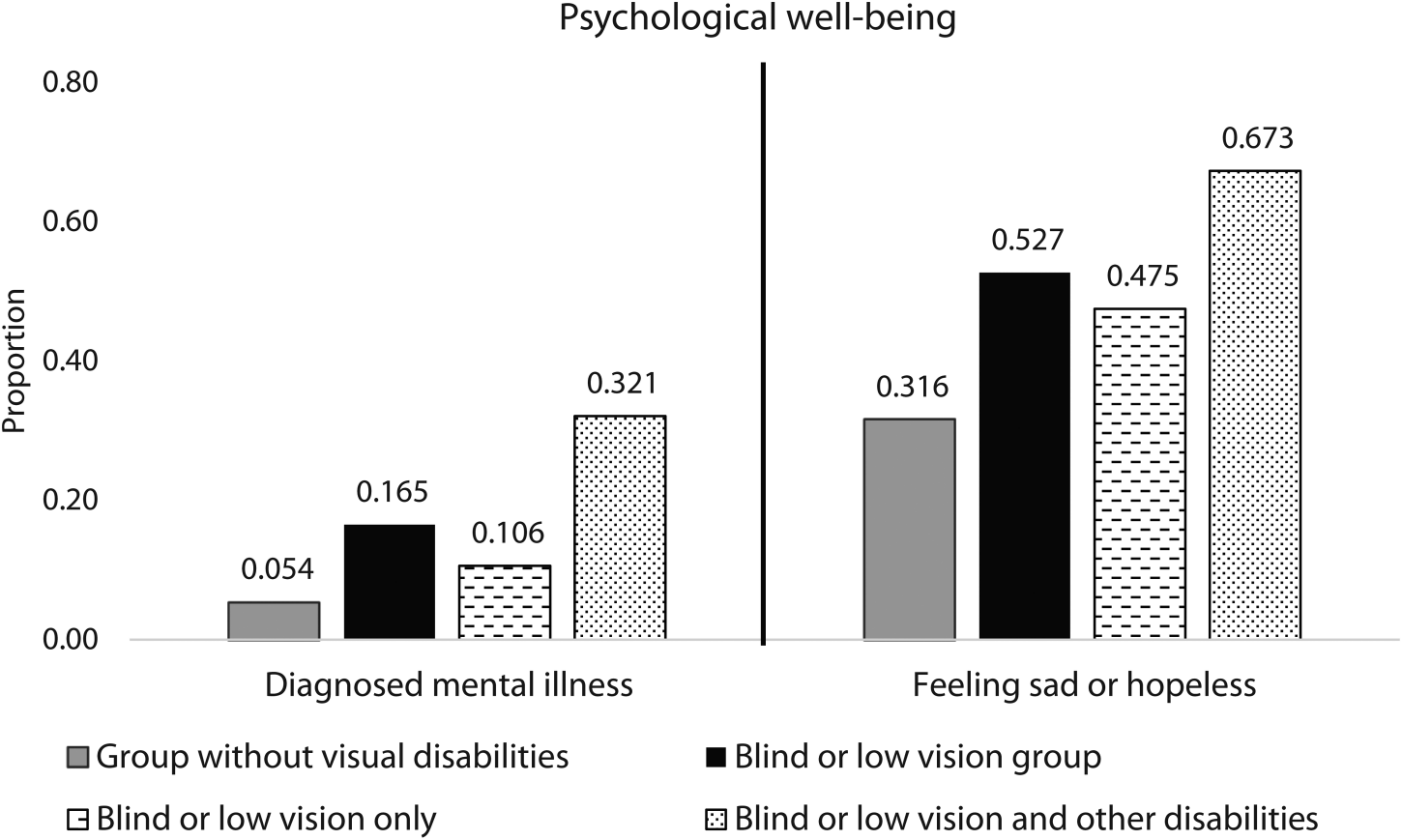
Proportion of Participants With Diagnosed Mental Illness and Those Feeling Sad or Hopeless

**Figure 5:**
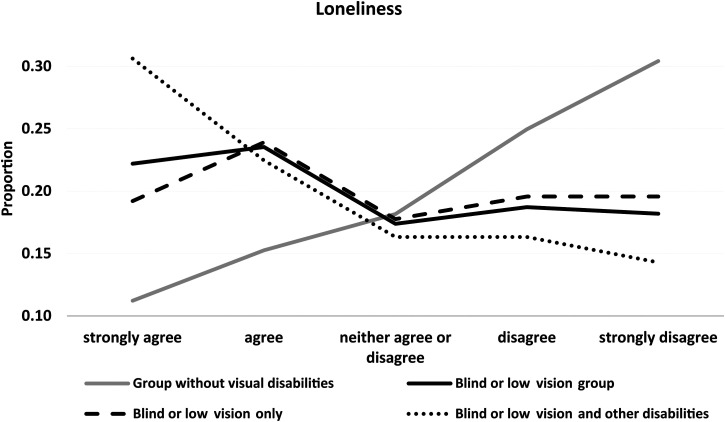
Proportion Breakdown of “I Often Feel Lonely” Responses

Compared to participants without visual disabilities, those who identify as blind or as having low vision reported much lower prevalence levels of a high subjective well-being (64.3% vs. 75.4% among participants without visual disabilities, 95% CI of difference −16.1% to −6.2%; see Table 2 along with Figure 1 for more details), a high life satisfaction (37.0% vs. 51.9%, 95% CI of difference −19.9% to −10.0%; see Figure 2), and an excellent or good health (70.1% vs. 82.2%, 95% CI of difference −16.8% to −7.5%; see Figure 3). Canadian adolescents who identify as blind or as having low vision had much higher prevalence rates of diagnosed mental illness (16.5% vs. 5.4% among participants without visual disabilities, 95% CI of difference 7.4% to 14.7%; see Figure 4 for more details), depression (52.7% vs. 31.6%, 95% CI of difference 16.0% to 26.2%; see Figure 4), and loneliness (45.7% vs. 26.5%, 95% CI of difference 14.1% to 24.3%; see Figure 5). Results from the bivariate chi-square tests indicate that the well-being of Canadian adolescents who are blind or have low vision differed significantly from that of their peers without visual disabilities (*p* < .0001). These differences were still statistically significant after adjusting for confounding variables age, gender identity, racial or cultural background, subjective socioeconomic status, and family structure.

A lower proportion of participants who are blind or have low vision and additional disabilities reported a high subjective well-being (55.9% vs. 67.2% among participants with visual impairments only, 95% CI of difference −22.8% to 0.3%; see Table 3 along with Figure 1 for more details). Additionally, a significantly smaller proportion reported a high life satisfaction (22.3% vs. 41.9%, 95% CI of difference −29.8% to −9.4%; see Figure 2), while only 59.4% reported having good or excellent health (vs. 73.9% among participants with visual impairments only, 95% CI of difference −25.3% to −3.6%; see Figure 3). In terms of psychological well-being, young blind or low vision individuals with multiple disabilities reported much higher prevalence levels of diagnosed mental illness (32.1% vs. 10.6% among participants with visual impairments only, 95% CI of difference 12.0% to 30.9%; see Figure 4 for more details), depression (67.3% vs. 47.5%, 95% CI of difference 8.9% to 30.9%; see Figure 4), and feelings of loneliness (53.1% vs. 43.1%, 95% CI of difference −1.5% to 21.4%; see Figure 5). No significant differences in proportions were observed between the two groups regarding high subjective well-being (*p* = .0998) or feelings of loneliness (*p* = .4957). Differences in life satisfaction, self-rated health, and mental health were still statistically significant after adjusting for the confounders.

## Discussion

Our study sought to explore the relative well-being of Canadian adolescents who are blind or have low vision. Our results affirm that Canadian adolescents who identify as being blind or as having low vision report lower well-being compared to their peers without visual disabilities. Importantly, the negative effect of blindness or low vision on well-being was significantly more pronounced among adolescents with multiple disabilities.

This study adds to the growing evidence that identifying as a person who is blind or has low vision is consistently associated with lower levels of subjective and psychological well-being. We found that participants with visual disabilities reported significantly higher prevalence levels of depressive symptoms and perceived themselves as lonelier than their peers without visual disabilities. Over half of the participants with visual disabilities indicated feeling so sad or hopeless almost every day for at least 2 consecutive weeks that they discontinued some of their regular activities. These results highlight the crucial need to address the subjective and psychological well-being of adolescents who are blind or have low vision and emphasize the importance of implementing targeted interventions to improve their well-being.

The lower levels of life satisfaction and poorer perceptions of health reported by Canadian adolescents who identify as being blind or as having low vision, compared to their peers without visual disabilities, highlight important disparities in well-being. These challenges may be further exacerbated by a higher likelihood of loneliness and poorer psychological well-being among adolescents who are blind or have low vision.

Similar findings have been reported in studies on children and adolescents with visual disabilities, which highlight lower life satisfaction, increased experiences of loneliness, and mental health challenges in this population (e.g., Boadi-Kusi et al., 2023; Chiracu & Buică-Belciu, 2023; Habib & Irshad, 2018). These results are also consistent with research on adults with visual impairments (e.g., Brunes et al., 2019; Schuster et al., 2018).

Our findings further indicate that, compared to adolescents with visual disabilities only, those with multiple disabilities such as autism, deafness, learning or physical disabilities, in addition to being blind or have low vision experience even worse well-being. Fewer Canadian adolescents who are blind or have low vision with additional disabilities report high levels of life satisfaction and excellent health, while a higher proportion of this population experience high rates of depressive symptoms—although both groups reported similar levels of loneliness in our study.

To promote positive development among adolescents who are blind or have low vision, it is essential to integrate measures of subjective well-being into social policies and intervention strategies (Casas & Frønes, 2020). Expanding access to specialized psychologists or counselors trained to support adolescents with visual impairments and multiple disabilities can help address their unique mental health needs. Additionally, fostering inclusive school activities that encourage meaningful participation can improve social support systems, reduce isolation, and improve the overall well-being.

### Limitations

Limitations of this study warrant consideration. It is important to acknowledge that these data are cross-sectional, which limits our ability to make causal inferences. Second, this study was conducted before the COVID-19 pandemic. Although the timing of the study limits its ability to reflect current trends, the well-being outcomes observed have likely worsened during and after the pandemic (e.g., Heinze et al., 2021; Ito et al., 2024; Tantirattanakulchai et al., 2023). Future research should build on the findings of this study using more recent data to examine postpandemic changes. Third, relying on self-reported data can potentially lead to response bias. Nevertheless, anonymous self-administered questionnaires have demonstrated reliability in disclosing sensitive information (Fridh et al., 2018). Finally, the study lacked information on the severity of reported disabilities, potentially limiting the generalizability of our findings.

## Conclusion

Our findings are consistent with previous research in this area and suggest that visual disability alone is a significant risk factor for poor well-being outcomes. We showed that the presence of multiple disabilities can exacerbate the already lower levels of well-being experienced by adolescents who are blind or have low vision. This research provides an opportunity to engage with health care professionals and policymakers to address the unique needs of visually disabled adolescents. Targeted interventions by educators, policymakers, and communities are urgently needed to improve their overall well-being.
